# Establishment of neural stem cell culture from the central nervous system of the Iberian ribbed newt *Pleurodeles waltl*

**DOI:** 10.1111/dgd.12820

**Published:** 2022-11-17

**Authors:** Ryohei Seki‐Omura, Shinichi Hayashi, Souichi Oe, Taro Koike, Yousuke Nakano, Yukie Hirahara, Susumu Tanaka, Masaaki Kitada

**Affiliations:** ^1^ Department of Anatomy, Faculty of Medicine Kansai Medical University Hirakata Japan; ^2^ Present address: Faculty of Nursing Kansai Medical University Hirakata Japan; ^3^ Present address: Faculty of Nursing and Nutrition University of Nagasaki Nagasaki Japan

**Keywords:** central nervous system, neural stem cell, neurosphere, newt, regeneration

## Abstract

Urodele amphibians have exceptional regeneration ability in various organs. Among these, the Iberian ribbed newt (*Pleurodeles waltl*) has emerged as a useful model organism for investigating the mechanisms underlying regeneration. Neural stem cells (NSCs) are an important source of regeneration in the central nervous system (CNS) and their culture method in vitro has been well established. NSCs form spherical cell aggregates called neurospheres and their formation has been demonstrated in various vertebrates, including some urodele species, but not in *P. waltl*. In this study, we reported neurosphere formation in brain‐ and spinal cord‐derived cells of post‐metamorphic *P. waltl*. These neurospheres showed proliferative activity and similar expression of marker proteins. However, the surface morphology was found to vary according to their origin, implying that the characteristics of the neurospheres generated from the brain and spinal cord could be similar but not identical. Subsequent in vitro differentiation analysis demonstrated that spinal cord‐derived neurospheres gave rise to neurons and glial cells. We also found that cells in neurospheres from *P. waltl* differentiated to oligodendrocytes, whereas those from axolotls were reported not to differentiate to this cell type under standard culture conditions. Based on our findings, implantation of genetically modified neurospheres together with associated technical advantages in *P. waltl* could reveal pivotal gene(s) and/or signaling pathway(s) essential for the complete spinal cord regeneration ability in the future.

## Graphical abstract

We report the formation of spherical cell aggregates similar to neurospheres *in vitro* from the brain and spinal cord of post‐metamorphic *Pleurodeles waltl*. These aggregates expressed several marker proteins of neural stem cells, and cells in the aggregates could differentiate into neurons, astrocytes, and oligodendrocytes *in vitro*. Based on our findings, we conclude that the observed spherical cell aggregates could be regarded as neurospheres from the central nervous system of *P. waltl*.

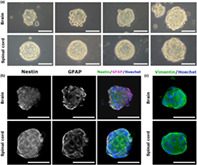

## 1 INTRODUCTION

Amphibians are good model organisms for the study of vertebrate regeneration. In particular, urodeles, such as axolotls and newts, have extraordinary regeneration abilities. Several organs, including the heart, limb, eye, jaw, tail, brain, and spinal cord, can regenerate after injury or amputation, although this ability is not identical among urodeles (Joven et al., 2019). One of the most established models is *Pleurodeles waltl*, for which genomic and transcriptomic data are now available (Elewa et al., 2017; Matsunami et al., 2019; Woych et al., 2022). Moreover, procedures for CRISPR/Cas9‐mediated genome editing and transgenesis have also been established (Cai et al., 2019; Elewa et al., 2017; Hayashi et al., 2013, 2019; Joven et al., 2018; Suzuki et al., 2018). The Mexican axolotl (*Ambystoma mexicanum*) is a neotenic species and another excellent model for studying organ regeneration (Joven et al., 2019). In contrast, *P. waltl* is a metamorphic species, suggesting that it could exhibit different regeneration outcomes compared to *A. mexicanum* because embryonic or larval animals have higher regeneration ability than post‐metamorphic animals.

Neural stem cells (NSCs), which have the potential to self‐renew and differentiate into neurons and glial cells, contribute to regeneration of the central nervous system (CNS) in urodeles as well (Berg et al., 2010, 2011; Kirkham et al., 2014; Parish et al., 2007; Urata et al., 2018). Culturing NSCs in vitro gives rise to sphere‐shaped cell aggregates called neurospheres, which were first reported in a study using adult mice (Reynolds & Weiss, 1992). In urodeles, neurosphere formation has been reported in *A. mexicanum* and red‐spotted newts (*Notophthalmus viridescens*), and transplantation of neurospheres into the injured spinal cord of axolotl demonstrated implanted neurospheres contribute to regeneration of the spinal cord (Mchedlishvili et al., 2012; Sun et al., 2018). However, neurosphere formation in *P. waltl* has not been reported.

In the present study, we investigated the possibility of neurosphere formation in *P. waltl* and examined the expression of NSC markers. We show that both brain‐ and spinal cord‐derived cell aggregates can be generated using a standard neurosphere culture method. The morphology of cell aggregates differed depending on their origin, implying that NSCs in the brain and spinal cord have different properties in *P. waltl*. We also demonstrate that cell aggregates derived from the spinal cord can differentiate into neurons, astrocytes, and oligodendrocytes. The success of neurosphere formation in *P. waltl*, an excellent regeneration model, would offer new opportunities to investigate the mechanisms of neural repair.

## 2 MATERIALS AND METHODS

### 2.1 Animals

The Iberian ribbed newts (*P. waltl*) were procured from the Hiroshima University Amphibian Research Center and kept in the laboratory as described previously (Hayashi et al., 2013). Post‐metamorphic individuals (4–7 months old) were used in this study. All animal treatments were performed in accordance with the institutional animal care and use committee guidelines of Kansai Medical University (approval numbers: 21‐050(1), 22‐074).

### 2.2 Cell culture

Neurosphere cultures were performed as previously described (Hameed & Simon, 2015; Kirkham et al., 2014). Briefly, animals were anesthetized with 0.05%–0.075% MS‐222 (Sigma, St. Louis, MO, USA) followed by dissection and collection of cerebral hemispheres and the spinal cord between the forelimb and hindlimb levels. Tissues were incubated in L‐15 medium (Thermo Fisher Scientific, Waltham, MA, USA) containing 15 U/ml papain (Sigma), 40 μg/ml DNase I (Sigma), and 1 mg/ml ovomucoid (Worthington Biochemical, Lakewood, NJ, USA) for 1 h at room temperature (RT). The reaction was terminated by adding equal volumes of L‐15 medium containing 2 mg/ml ovomucoid and 40 μg/ml DNase I. The samples were centrifuged at 80 ×*g* for 5 min and supernatant was discarded. The cells were dissociated by trituration with a 1‐ml pipette in fresh L‐15 medium for 2 min. The resultant cell suspension was filtered through a 40‐μm cell strainer followed by centrifugation at 80 ×*g* for 5 min. Next, 2 ml of expansion medium (DMEM/F‐12 with GlutaMAX™ supplement [Thermo Fisher] containing 2% B27 [Thermo Fisher], 20 ng/ml basic fibroblast growth factor [bFGF] [R&D Systems, Minneapolis, MN, USA], 20 ng/ml epidermal growth factor [EGF] [R&D Systems], and penicillin–streptomycin [FUJIFILM Wako, Osaka, Japan]) was added to the pellet, and the resultant cell suspension was transferred to a 35‐mm non‐coated dish. Cells from the brain of one animal were seeded at a density of 1–2 × 10^5^ cells/ml. Cells from the spinal cord could not be counted because of uncontrolled contamination of tissue‐derived debris. Thus, we applied spinal cord cells derived from one animal for culture without counting. Cells were incubated at 27°C with 2% CO_2_. Half of the expansion medium was changed once or twice a week. Neurospheres cultured for 2–4 weeks were used for the subsequent analyses. All experiments were performed in a primary culture system without any passage. For differentiation, neurospheres were plated on a poly‐L‐lysine (Sigma)‐coated cover glass in each well of a four‐well plate. The next day, expansion medium was replaced with a medium without bFGF or EGF. After 14 days, the differentiated cells were used for subsequent analyses. All media were diluted to 66% (v/v) with sterile water (FUJIFILM Wako) to adjust to the amphibian osmolarity.

### 2.3 Immunostaining

Immunocytochemistry was performed as described previously (Hameed & Simon, 2015) with some modifications. For spherical cell aggregates (SCAs), up to 75% of each solution was removed at every step to prevent loss due to aspiration. First, cells were fixed with 4% paraformaldehyde in phosphate‐buffered saline (PBS) for 15 min at RT, followed by washing with PBS thrice. Cells were permeabilized with PBS containing 0.5% Triton X‐100 (PBST) for 15 min and then blocked with 1% bovine serum albumin (BSA) in PBST for 30 min. The primary antibody reaction was performed overnight at 4°C. The following primary antibodies were used in this study: rabbit polyclonal anti‐phospho‐histone H3 (PHH3) (Ser3) (1:600–1000, 06‐570, Merck Millipore, Burlington, MA, USA), goat polyclonal anti‐Nestin (1:1000, AF2736, R&D Systems), mouse monoclonal anti‐glial fibrillary acidic protein (GFAP) (1:1000, sc‐33673, Santa Cruz Biotechnology, Santa Cruz, CA, USA), rabbit polyclonal anti‐GFAP (1:1000, Z0334, Dako, Glostrup, Hovedstaden, Denmark), goat polyclonal anti‐Vimentin (1:1000, V4630, Sigma), mouse monoclonal anti‐neuron‐specific beta‐III tubulin (clone TuJ‐1, 1:4000, MAB1195, R&D Systems), and mouse monoclonal O4 antibody (1:500, MAB345, Merck Millipore). After washing with PBS thrice, cells were incubated with 1% BSA in PBST for >10 min, followed by incubation with Cy3‐, Alexa Fluor 488‐, or 594‐conjugated secondary antibodies (1:1000 each) for 2 h at RT. After washing with PBS, cells were incubated with 2.5–5.0 μg/ml Hoechst 33258 in PBS for 30 min at RT, followed by washing with PBS thrice. Finally, cells were mounted with a solution containing Hoechst 33258, and fluorescence images were obtained using an FV3000 confocal laser scanning microscope (Olympus, Tokyo, Japan). For homology comparison of GFAP and beta‐III tubulin proteins between *P. waltl* and mouse/human, protein alignment was performed using the database iNewt (https://www.nibb.ac.jp/imori/main/).

## 3 RESULTS AND DISCUSSION

**FIGURE 1:**
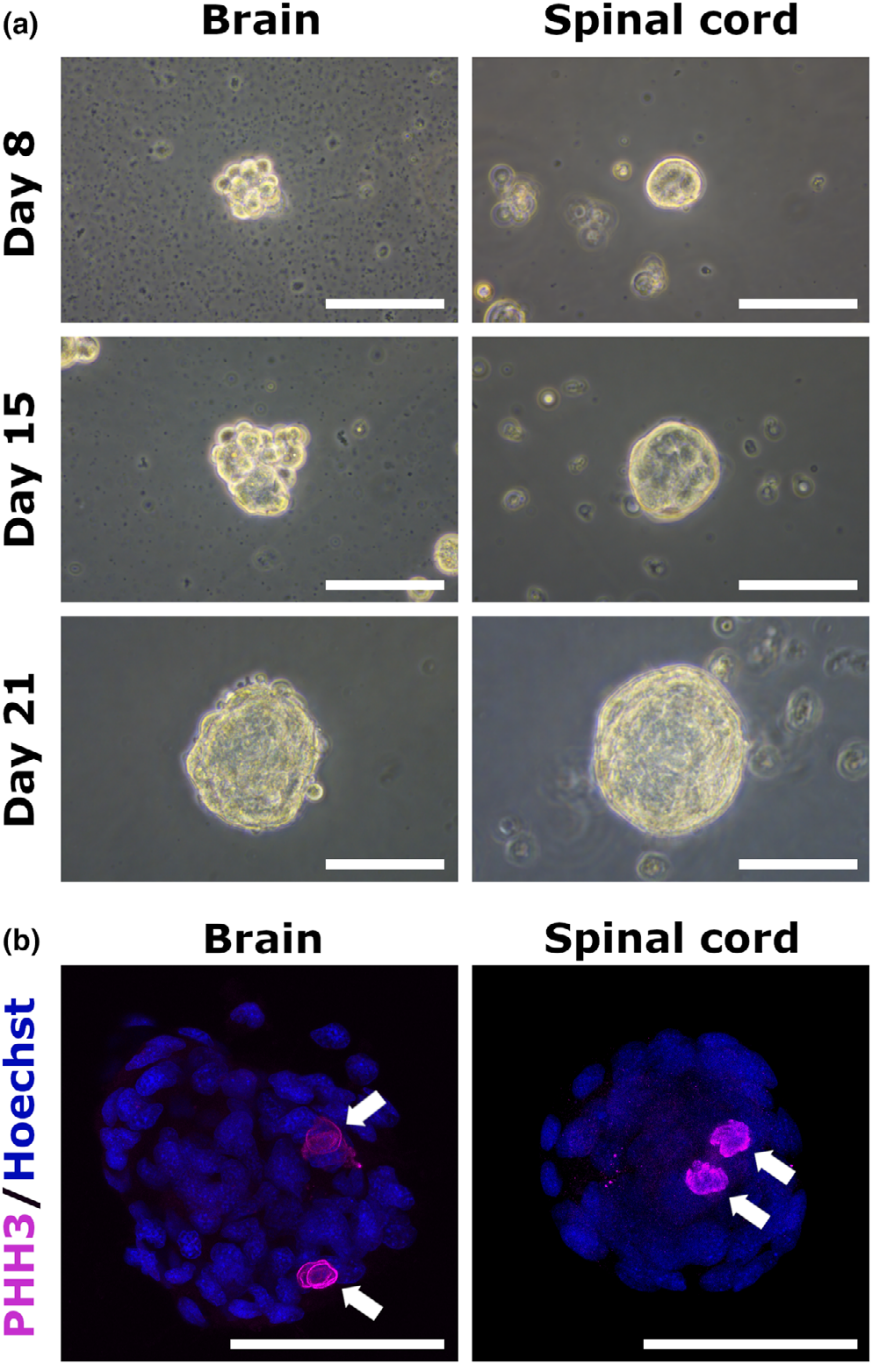
Spherical cell aggregate (SCA) formation of cells derived from the brain and spinal cord. (a) Brain‐ and spinal cord‐derived SCAs cultured for 8, 15, and 21 days. Similar results were obtained in six independent experiments. (b) Phospho‐histone H3 (PHH3)‐positive proliferative cells (magenta, indicated by arrows) were found in both brain‐ and spinal cord‐derived SCAs. Nuclei were stained with Hoechst 33258 (blue). Scale bars represent 100 μm.

The neurosphere formation in culture demonstrates the presence of NSCs in source tissue (Reynolds & Weiss, 1992). Therefore, we examined the neurosphere formation ability of cells isolated from the brain and spinal cord of *P. waltl*. SCAs were successfully generated from the brain‐ and spinal cord‐derived cells. The SCAs in culture were ~25 μm in diameter by day 8, and reached ~100 μm by day 21 (Figure 1a). The proliferative ability of SCAs was examined by immunostaining with an antibody for the mitosis marker PHH3, and some of them were found to be immunoreactive for PHH3 (Figure 1b), indicating that these SCAs with different neural origin (brain and spinal cord) have proliferative activity.

**FIGURE 2:**
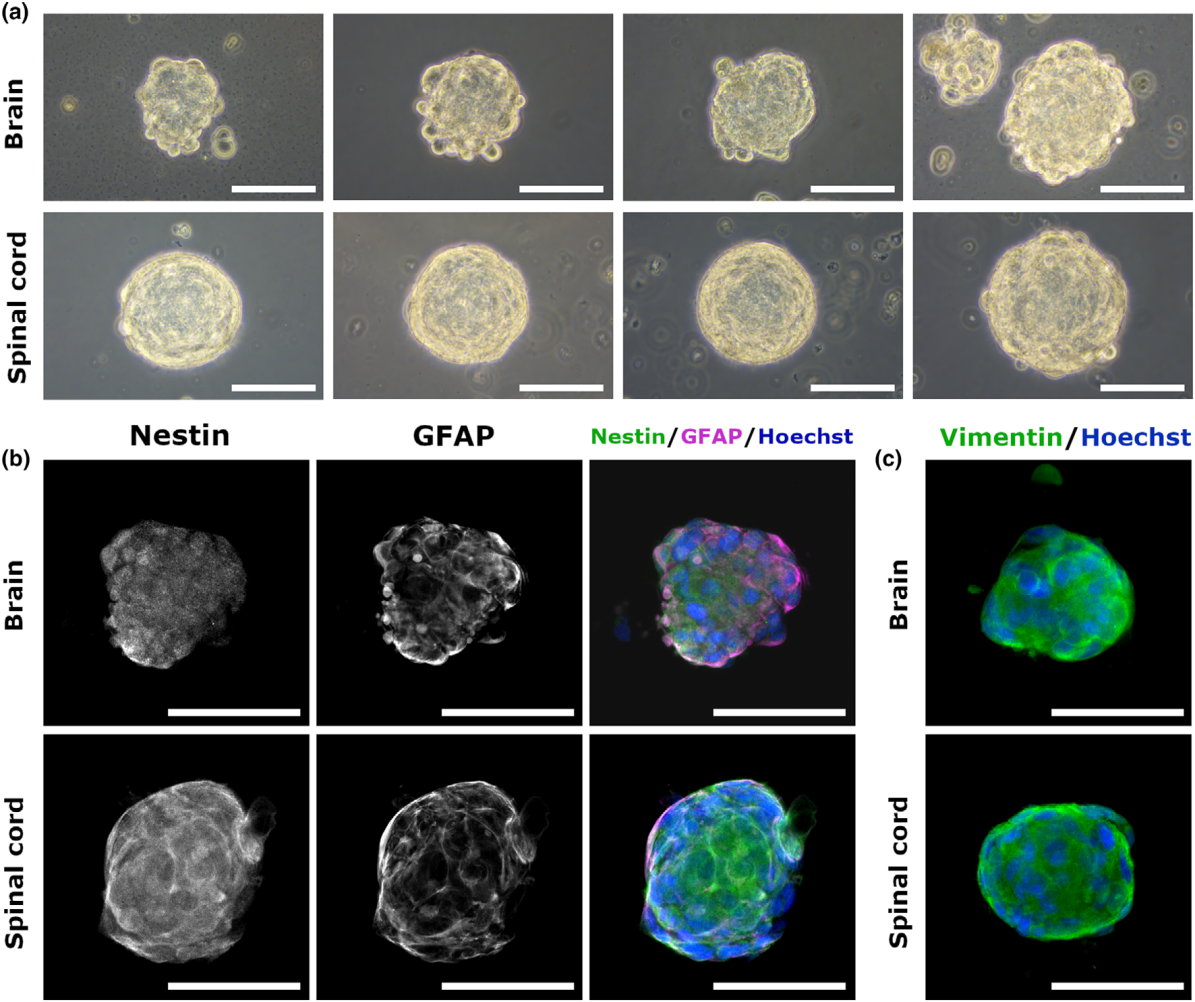
Morphological and immunocytochemical characterization of brain‐ and spinal cord‐derived spherical cell aggregates (SCAs). (a) SCAs derived from the brain and spinal cord and cultured for 17–26 days are shown. SCAs from the brain had a rugged surface, whereas those from the spinal cord had a smooth surface. (b) Immunostaining for Nestin (green) and GFAP (magenta). These two cell lineage marker proteins were expressed in both brain‐ and spinal cord‐derived SCAs. (c) Vimentin was also expressed in both SCAs (green). Nuclei were stained with Hoechst 33258 (blue). Scale bars represent 100 μm.

The analysis of reproduced cell aggregates from distinct CNS tissues revealed that the morphology of SCAs varied according to their origin. While brain‐derived SCAs had rough surfaces, SCAs derived from the spinal cord had smooth surfaces (Figure 2a). To characterize the cells in SCAs, expression of NSC markers was analyzed by immunostaining. Nestin, a marker for NSCs, was expressed in all SCAs, with a comparable expression pattern in both brain‐ and spinal cord‐derived SCAs (Figure 2b). GFAP, which is expressed in radial glial cells and astrocytes, was also detected in both SCAs (Figure 2b). In addition, another NSC marker, Vimentin, was highly expressed in SCAs regardless of their origin (Figure 2c). Considering their proliferative activity (Figure 1b), these results suggest that cells in SCAs resemble NSCs.

**FIGURE 3:**
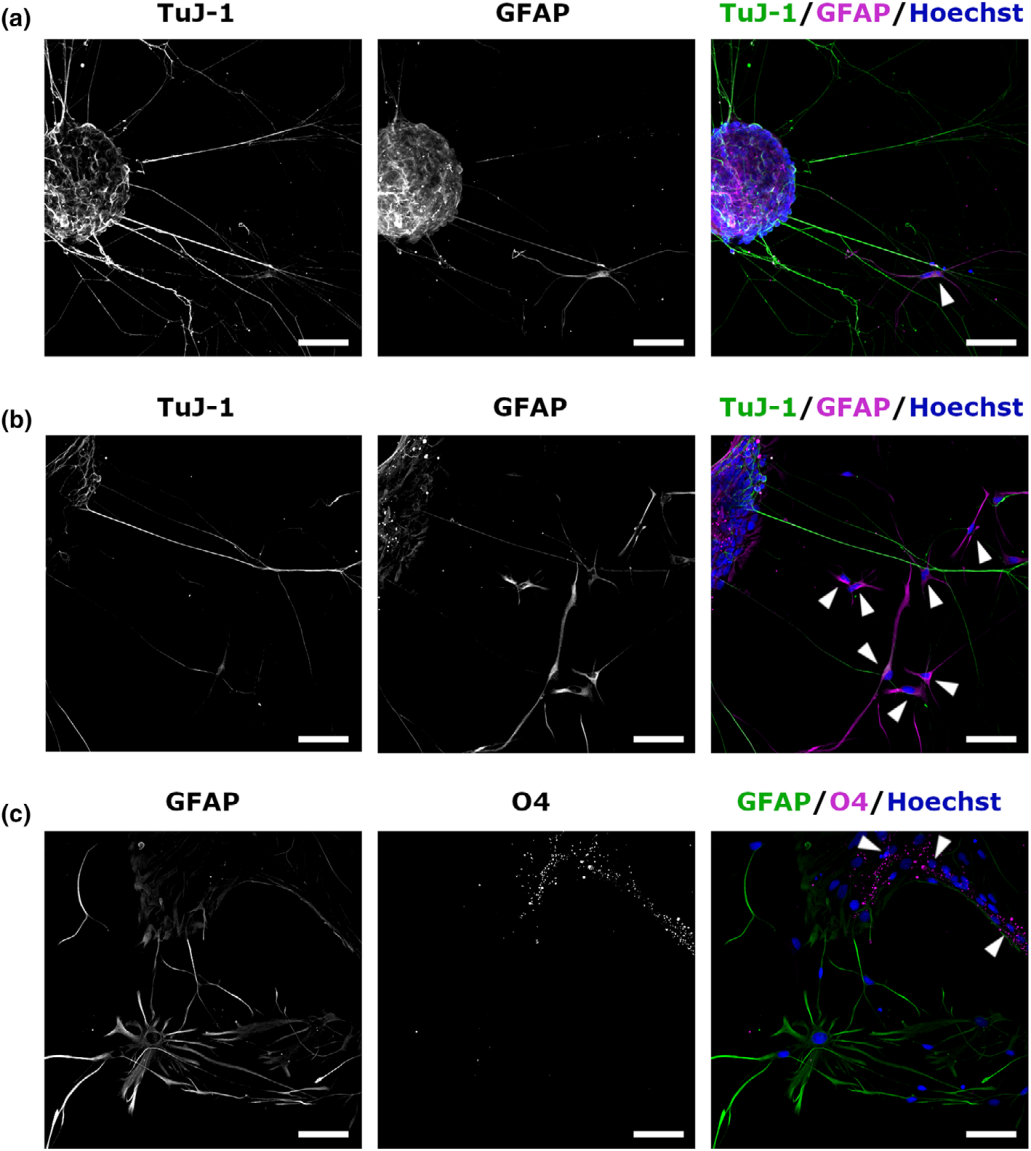
Differentiation of spherical cell aggregate (SCA)‐derived cells. SCAs derived from the spinal cord were plated on poly‐L‐lysine‐coated glass and cultured for 14 days to induce differentiation. (a, b) Immunocytochemistry for beta‐III tubulin (TuJ‐1) and GFAP. Neurons positive for TuJ‐1 (green) were also observed. The expression of GFAP (magenta) indicates differentiation into astrocytes (arrowheads). In some cases, SCAs generated a few astrocytes (a), whereas most SCAs generated many astrocytes (b). (c) Immunocytochemistry with anti‐GFAP and O4 antibodies. Arrowheads indicate O4‐positive oligodendrocytes (magenta) that were GFAP‐negative (green). Nuclei were stained with Hoechst 33258 (blue). Scale bars represent 100 μm.

The potential to differentiate into neurons and glial cells is another property of NSCs. We investigated this ability by inducing the differentiation of spinal cord‐derived SCAs in a medium without bFGF or EGF under adherent culture conditions. After 14 days, differentiating adherent cells were immunostained with specific antibodies against neurons and glial cells. The differentiation into neurons and astrocytes was confirmed by the expression of beta‐III tubulin and GFAP, respectively (Figure 3a,b). However, the frequency of GFAP expression varied; some SCAs generated a few (Figure 3a, arrowheads), while others gave rise to many GFAP‐positive cells (Figure 3b, arrowheads). Furthermore, cells generated from spinal cord‐derived SCAs were positive for O4 but negative for GFAP (Figure 3c, arrowheads), indicating differentiation into oligodendrocytes. These results suggest that spinal cord‐derived SCAs of *P. waltl* include cells having a similar differentiation potential to that of NSCs.

The epitopes of monoclonal antibodies against GFAP and beta‐III tubulin used in this study have not been clarified yet. Instead, we analyzed the homology of these proteins between *P. waltl* and mouse/human using the iNewt database (see Materials and methods). The amino acid identity of putative GFAP of *P. waltl* and mouse/human is 64%/64% for isoform 1 (the longest isoform). Also, the amino acid identity of putative beta‐III tubulin of *P. waltl* and mouse/human is 93%/93% for isoform 1 (the longest isoform).

In the present study, we cultured NSCs from the CNS of *P. waltl* and investigated their characteristics in vitro. Under standard neurosphere culture conditions (Hameed & Simon, 2015; Kirkham et al., 2014), the brain‐ and spinal cord‐derived cells formed SCAs, which exhibited proliferative activity, expressed typical NSC markers (Figures 1 and 2), and differentiated into neurons and glial cells (Figure 3). These results are consistent with those of previous studies, in which neurospheres were generated from other salamander species (Kirkham et al., 2014; Mchedlishvili et al., 2012; Sun et al., 2018). One exception is that cells in neurospheres derived from the spinal cord of *A. mexicanum* did not exhibit differentiation into oligodendrocytes in the absence of sonic hedgehog (Shh) agonist and platelet‐derived growth factor (PDGF) (Mchedlishvili et al., 2012), which contrasts with our results. This difference might reflect the species‐specific property of neurospheres, or it might be just due to differences in procedures for the detection of oligodendrocytes: O4 antibody and anti‐myelin basic protein were respectively used in the present and previous studies. Our results indicate that the neurosphere culture technique based on previous reports (Hameed & Simon, 2015; Kirkham et al., 2014) was suitable to culture and expand NSCs from the CNS of *P. waltl*. Theoretically, the self‐renewal property of NSCs is demonstrated by repeated neurosphere formation from a single cell to show the differentiation potential into neurons and glial cells from neurospheres in each generation. Therefore, further studies are required to confirm this property.

The surface morphology of SCAs varied according to their tissue origin (Figure 2a), which implies that NSCs with different characteristics exist in the brain and spinal cord, although variations in their molecular expression were not addressed in this study. It is also unclear how the surface morphology could affect or reflect characteristics of NSCs. Regarding the surface morphology of cell aggregates derived from stem cells, several studies have shown that the surface morphology of the spheroid of glioma stem cells (GSCs) reflects their subtype, with a smooth surface indicating the less malignant proneural subtype and a rough surface indicating the more malignant mesenchymal subtype (see Figure 3a in Spinelli et al., 2018 and Figure 1a in Garnier et al., 2018). Although the relationship between the surface morphology and characteristics of GSCs have not been fully clarified yet, it may be possible that the differences in the surface morphology of neurospheres derived from the brain and spinal cord of *P. waltl* also reflect the differences in the characteristics such as the potential of differentiation and proliferation. Consistent with our findings, a previous report identified 229 differentially expressed genes in mouse embryonic cortex‐ and spinal cord‐derived neurospheres (Kelly et al., 2009). The properties of brain‐ and spinal cord‐derived neurospheres of *P. waltl* may also differ. For example, a previous study showed that cells from the tail spinal cord of *P. waltl* gave rise to neural crest lineage cells when transplanted into a newt tail regeneration model (Benraiss et al., 1997), suggesting that spinal cord‐derived NSCs might have higher differentiation potential than those from brain tissue. Thus, it would be interesting to investigate the potential of NSCs of *P. waltl* to differentiate into non‐neural lineages in the future. Alternatively, other factors might be important for the formation of the surface morphology of neurospheres. The spheroid morphology in a 3D liver spheroid model was shown to be highly dependent on the initial density of culturing cells and the number of each spheroid (Gaskell et al., 2016). They also demonstrated that the size of each spheroid did not correspond to the number of cells in each spheroid: the cell numbers in spheroids sometimes differ even if the sizes of spheroids are equivalent. In our study, because the number of spinal cord‐derived cells could not be precisely counted we did not unify the initial density of cells in culturing (see Materials and methods). Also, we did not count the number of cells constituting each neurosphere. Therefore, we cannot rule out the possibility that the initial density of cells in the culture and/or the number of cells in each neurosphere influence the surface morphology of neurospheres.

Recently, rapid technological innovations have made *P. waltl* an excellent model organism, especially for regeneration research. In the present study, we reported the formation of neurosphere‐like cell aggregates from brain‐ and spinal cord‐derived cells of this increasingly useful animal. Many studies using mammalian models have suggested that neurosphere implantation is a potential therapeutic strategy for patients with spinal cord injury (Chow et al., 2000; Lee‐Kubli & Lu, 2015; Nakamura et al., 2005; Ramotowski et al., 2019; Wu et al., 2001). Similarly, neurospheres and/or NSCs implanted into the injured spinal cord after tail amputation were shown to contribute to regeneration in axolotl (Mchedlishvili et al., 2012; Sun et al., 2018). Using *P. waltl* as a spinal cord injury model and associated technical advantages, implantation of genetically modified neurospheres could reveal pivotal gene(s) and/or signaling pathway(s) essential for the complete spinal cord regeneration ability. These findings could provide insights for future clinical applications.

## AUTHOR CONTRIBUTIONS

Ryohei Seki‐Omura and Masaaki Kitada designed the study. Ryohei Seki‐Omura performed the experiments and drafted the manuscript. Shinichi Hayashi, Souichi Oe, Taro Koike, Yousuke Nakano, Yukie Hirahara, Susumu Tanaka, and Masaaki Kitada provided suggestions for appropriate experiments and provided critical comments to improve the manuscript. All authors have read and approved the final version of the manuscript.
